# Cone-beam computed tomography (CBCT) analysis of maxillary sinus septa in Indians

**DOI:** 10.6026/97320630018251

**Published:** 2022-03-31

**Authors:** T.Sukumar Archana, A.Rangaiah Vinod Kumar, Akshay Shetty, Nida Ahmed, Bhavana T Veerabasvaiah, Fazeel Ahmed

**Affiliations:** 1 Department of Oral and Maxillofacial surgery, AECS Maaruti College of Dental Sciences and Research Renter, Bannerghata road, Bangalore 560076; 2 Oral Medicine and Radiology, Oral 3D Diagnostic centre Bangalore 560076; 3 Department of Oral Medicine and Radiology, Sri Rajiv Gandhi College of Dental Sciences & Hospital, Cholanagar, Bangalore 560032; 4 Healing Care medical center, Kulhudhuffushi Island, Maldives

**Keywords:** Cone Beam Computed Tomography (CBCT), Implant, Maxillary sinus, Septa

## Abstract

It is of interest to assess the presence of maxillary sinus septae in patients undergoing implant treatment using Cone Beam Computed Tomography (CBCT).This retrospective study evaluated CBCT scans of 99 patients who opted for implant placement. A total of 198 sinuses were analyzed. The cases were divided into two group's namely edentulous group and non-edentulous groups. The location of septa was divided for analysis into 3 regions namely, the anterior (1st and 2nd premolar), middle (1st and 2nd molar) and posterior (behind 2nd molar) regions. Out of 198 sinuses assessed 15 sinuses had septa. It was more common in males. Mean height of septa was 7.7mm. It was more commonly seen in the middle region (1st and 2nd molar). All of the septa were partial in nature. Septa were common on the right side. It was absent in the edentulous group. To conclude this study showed low prevalence of septa in patients who were assessed as a part of pre-operative planning for implant placement. Modified sinus lift procedures were completed for placement of bone grafts in patients with septa,. This reduced the chances of membrane perforation and increases the chances of better outcomes. CBCT with its low cost and high resolution is useful for assessing the sinus.

## Background:

Implants have been increasing used for rehabilitation in the posterior edentulous space. The biggest challenge in posterior maxilla is reduced bone height which is limited by presence of maxillary sinus [1]. Placement of dental implants in such patients requires procedures such as alveolar bone grafting and maxillary sinus lifts [2]. Preoperative evaluation of the maxillary sinuses prior to any augmentation is essential for minimizing postoperative complications and increasing the likelihood that the procedure will succeed [3,4]. The design of the lateral window during sinus lift procedures are based on the presence and size of maxillary sinus septa [5]. Maxillary sinus has been described as having inverted Gothic arch structure. It has been seen to arise from inferior and lateral walls of maxillary sinus [6]. With its low cost, low radiation dose, and high spatial resolution, CBCT is becoming the modality of choice for evaluating potential implant sites, especially in complex cases that require three-dimensional views of the area of interest [4]. Therefore, it is of interest to assess the presence of maxillary sinus septae in patients undergoing implant treatment using Cone Beam Computed Tomography (CBCT).

## Materials and Methods:

98 patients who were planned for pre implant surgery were referred for CBCT examination of the maxilla. The examinations were performed using cs9300. The tomography specifications were: tube potential90 (kV), tube current10 (mA), reconstruction time (s), and Voxel size 180 micron (mm) 0, scan time 16(s). DICOM images were assessed in all the three planes ieaxial, sagittal and coronal. CBCT datasets of patients were selected to include only those patients where the dataset showed a complete maxillary sinus and maxillary sinus floor (Figure 1 - see PDF).

## Results:

The study population consisted 99 patients with mean age of 51yrs Table 1(see PDF). Total of 198 sinuses were assessed. 6 of the patients were edentulous. 15 sinuses had septa. It should be noted that only 1 septa is present in the sinus per case. Overall mean height of the septa was 7.7mm. Table 2(see PDF) One patient had septa in both the sinuses.9 sinuses had septa in right side with mean height of 7.94mm. 6 sinuses had septum in left with mean of 7.8 mm (Figure 2 - see PDF). Most common location of the septa was in the middle. Only three septa were seen anteriorly No septa were seen in edentulous cases. Septa were more common in males than in females. All the septa were partial septa, no complete septa was seen in this study.

## Discussion:

The management of deficient bone height in posterior maxilla presents a challenge to the trained maxillofacial surgeon as well as the young implantologist. The advent of new techniques like zygoma, cortical impants and well established sinus lift procedures depend on accurate measurements to ensure optimal treatment outcomes. Assessment of maxillary sinus is extremely important prior to implant placement because of numerous anatomical variations present within the sinus. This may require modification in sinus lift procedures routinely carried out to increase the height of the alveolar bone. Underwood was the first to describe maxillary sinus septa.6 number of hypothesis has been suggested as the etiology of septa. Underwood suggested that bony septa are thin, shattered and sharp ended structures that are related to tooth development. They arise from the floor between the area of two adjacent teeth and divide the sinus into 3 compartments: anterior, middle and posterior [6, 7]. Septa was also considered as a kind of reinforcement that holds the volume and shape of maxillary sinus and could be due to disharmony during the growth of bone surface sutures in the alveolar process and maxillary Sinus [8,9]. Neivert proposed the idea that septa were derived from the finger like projections produced during embryological out-pouching of the ethmoid infundibulum in cases when the clinging wall did not resorb [10]. It is known that irregular process of sinus floor atrophy in different regions created bony crest, and the resulting septa was called as secondary septa [11]. The primary septa, formed during the development of bony head structures and the head's height, increase with the growing process of the head and secondary septa are formed after tooth loss in the process of maxillary alveolar atrophy [12]. Several authors have studied the prevalence of maxillary sinus septa and found 30 septa in 90 sinuses, demonstrating 33% prevalence and found 15 septa in 82 sinuses, demonstrating a prevalence of 18.3%. Also, reported 32 septa in 200 sinuses, demonstrating 16% prevalence [12] and reported 75 septa in 312 sinuses, demonstrating a prevalence of 24% [14].This study revealed 15 septa (7%) in 198 sinuses showing low incidence in the present study. The present study showed higher incidence in males but few studies have showed female predilection [16]. The prevalence of septa in the right sinus was higher than in the left sinus, at 60% (9/15) in the right, compared to 40% (6/15) in left, however the difference was small [17]. The prevalence of septa in both sinuses was low, at 6% (1/15). Studies have reported 72 septa in 312 sinuses, and found 39 in the left sinus and 36 in the right sinus showing higher incidence in the left side as opposed to ours [14]. Based on location the highest incidence in this study was found in the middle which was similar to the results of other studies [15,18]. The mean height was 7.4 mm in the present study. A mean height of septa between 6.4 and 12.7 mm. [7] and average heights between 2.5 and 6 mm can be reported [18]. Septa may be partial or complete. Complete septa divide the sinus in to two different cavities. In our study all the septa were partial septa. Similar results were seen in other studies.

## Conclusion:

Three dimensional imaging of the sinus plays a significant role in treatment planning. Presence of anatomic variations may warrant modification in the procedures for the better outcome of the implant. CBCT with its lower cost is useful for 3D imaging modality that could be routinely used as a part of treatment planning.
